# Soft Tissue Sarcoma Cancer Stem Cells: An Overview

**DOI:** 10.3389/fonc.2018.00475

**Published:** 2018-10-26

**Authors:** Katia C. Genadry, Silvia Pietrobono, Rossella Rota, Corinne M. Linardic

**Affiliations:** ^1^Division of Hematology-Oncology, Department of Pediatrics, Duke University Medical Center, Durham, NC, United States; ^2^Department of Hematology-Oncology, Bambino Gesù Pediatric Hospital, IRCCS, Rome, Italy; ^3^Department of Pharmacology & Cancer Biology, Duke University Medical Center, Durham, NC, United States

**Keywords:** stemness, cancer stem cells, sarcoma, epigenetic plasticity, developmental pathways, soft tissue sarcoma

## Abstract

Soft tissue sarcomas (STSs) are an uncommon group of solid tumors that can arise throughout the human lifespan. Despite their commonality as non-bony cancers that develop from mesenchymal cell precursors, they are heterogeneous in their genetic profiles, histology, and clinical features. This has made it difficult to identify a single target or therapy specific to STSs. And while there is no one cell of origin ascribed to all STSs, the cancer stem cell (CSC) principle—that a subpopulation of tumor cells possesses stem cell-like properties underlying tumor initiation, therapeutic resistance, disease recurrence, and metastasis—predicts that ultimately it should be possible to identify a feature common to all STSs that could function as a therapeutic Achilles' heel. Here we review the published evidence for CSCs in each of the most common STSs, then focus on the methods used to study CSCs, the developmental signaling pathways usurped by CSCs, and the epigenetic alterations critical for CSC identity that may be useful for further study of STS biology. We conclude with discussion of some challenges to the field and future directions.

## Introduction

Despite substantial therapeutic advances, cancer is still a significant cause of morbidity and mortality world-wide (1, 2). Solid tumors in particular show a complex mix of genomic subclones with different mutational signatures and cellular phenotypes. This “intratumoral heterogeneity” is thought to be the primary cause of therapeutic failure, followed by disease progression and relapse (3–6). To date, two opposing models have been offered to explain such tumor heterogeneity. The “stochastic” model considers it as a result of cellular natural selection (7). As such, every cell within a tumor has equivalent tumorigenic potential, and random mutations in individual tumor cells promote the selection of the fittest clone. Over time, additional advantageous mutations spawn genetically divergent subclones that independently maintain their malignant potential. By contrast, the cancer stem cell (CSC) model ascribes tumor establishment to a single, transformed, stem-like clone endowed with dysregulated and unlimited self-renewal that differentiates into less tumorigenic subclones to create a cellular “hierarchy” similar to that of normal tissue (8, 9).

CSCs, also known as “tumor-initiating cells” (TICs), were first identified in cancers of hematopoietic origin (10, 11), then in various solid tumors (12–17), where these cells show the ability to re-derive the original tumor heterogeneity when serially xenotransplanted in immunocompromised mice at very low number (8, 9). The importance of CSCs comes from their ability to constitute a small reservoir of drug-resistant cells, which overcome conventional chemotherapy due to their low rate of proliferation, thus driving tumor recurrence and metastasis (18). CSCs share several properties with non-transformed adult stem cells (SCs): multipotency, resulting in the possibility to differentiate into lineages of the embryonic layer of origin; self-renewal capacity; the ability to transition between quiescence, slow-cycling and active proliferation states; the expression of the embryonic stem cell transcription factors (TFs) OCT4, NANOG, SOX2, KLF4, and MYC; the expression of similar surface markers such as CD44, CD133, or of the intracellular enzyme aldehyde dehydrogenase (ALDH); enhanced protective mechanisms against apoptosis, DNA repair, and oxidative stress; activation of key developmental pathways; a preference for oxidative metabolism for energy production, and the expression of ATP-binding cassette (ABC) drug transporters (19). However, unlike normal SCs, CSCs are characterized by the dysregulation of those features and possess the ability to control stemness signals (5, 20).

Although the source of CSCs has been the center of investigation for many years, it remains a hotly debated topic. Reflecting the ongoing discourse, it has been suggested that the clonal evolution (stochastic) and the CSC (hierarchical) models are not necessarily mutually exclusive, as CSCs can originate not only from the malignant transformation of adult SCs (21–23) but also from mutated tumor cells that undergo a de-differentiation process in which they reacquire stem-like features in order to evolve into CSCs (24, 25). Both cell-autonomous (activation of stemness signaling pathways, epigenetic alterations, DNA damage, replicative stress) and non-cell-autonomous factors (immune system, tissue damage, signals from the microenvironment niche, therapy-induced genotoxic stress) seem to participate to the reprogramming [reviewed in Poli et al. (26)].

Soft tissue sarcomas (STSs) are uncommon malignancies of mesenchymal origin characterized by a high degree of heterogeneity in their genetic profile, histology and clinical features. They include several subtypes with onset in childhood, adolescence and even in the adult life, such as rhabdomyosarcoma (RMS), synovial sarcoma (SS), fibrosarcoma (FS), malignant peripheral nerve sheet tumor (MPNST), leiomyosarcoma (LMS), liposarcoma (LPS) and undifferentiated pleomorphic sarcoma (UPS). The prevalence of STS subtypes significantly changes from childhood (<20 years) through adolescence into adulthood representing about 5–6% of all childhood cancers and <1% of all adult malignancies (27). Various studies suggest that STSs originate from the malignant transformation of a primitive, multipotent mesenchymal stem cell (MSC), i.e., the multipotent precursor of mesodermal tissues including bone, skeletal muscle, adipose tissue, cartilage and tendon (28). It has been suggested that the same transformed MSC can give a particular subtype of STS depending on the vulnerability to subsequent mutations involving specific developmental pathways or, alternatively, in the genesis of an undifferentiated sarcoma (29, 30). However, gene expression studies showed that the signatures of several STSs were more similar to that of differentiated MSCs than of undifferentiated MSCs, suggesting that the development of STSs with distinct phenotypes and histological grades may reflect different differentiation stages of MSCs at the time of tumorigenesis initiation (31–33).

Contemporary therapies for STSs are multi-modal and include surgery, radiation and chemotherapy, although significant limitations are provided by their toxicity and partial responses. To date, the 5-year survival rate for patients with STS is about 60%, reflecting age, tumor type, stage and histologic grade, but it drops dramatically to 10–17% in high risk patients (34). Recently, STS tumor cells with stem-like properties have been identified, possibly explaining the heterogeneity that characterizes these cancers and suggesting that these cells might be responsible for relapse and metastasis.

CSC characteristics and their role in tumorigenesis has been studied and summarized for various solid tumors, including bone sarcomas (35–38). However, an overview on the role of CSCs in STS is lacking. This review summarizes the evidence for CSCs in STSs. The importance of CSC features for clinical anticancer interventions is also discussed.

## Evidence for CSCs in soft-tissue sarcomas

According to the type of genomic alteration, STSs can be classified into two main categories: (i) recurrent translocation-driven STSs, where reciprocal chromosomal translocations result in oncogenic fusion transcripts such as *PAX3-FOXO1* in alveolar RMS (ARMS), *SS18-SSX* in SS, *FUS-CHOP* in myxoid/round-cell LPS, and (ii) non-translocation driven STSs characterized by complex genetic changes such as amplifications/deletions in various chromosomal regions as observed in embryonal RMS (ERMS), FS, LMS, LPS and MPNSTs (39). Fusion-positive STSs are characterized by cells that are morphologically and molecularly similar with the fusion oncoprotein as the major driver of the malignancy. Conversely, fusion-negative STSs show a high degree of intra-tumor heterogeneity.

### Rhabdomyosarcoma (RMS)

RMS is the most common soft tissue sarcoma in children and young adults but can occur at any age (40, 41). RMS is thought to derive from myogenic precursors that lose the ability to differentiate into skeletal muscle despite the expression of the master key genes of skeletal muscle lineage (42). The two main histopathologic subtypes are ARMS and ERMS. ARMS is associated with a poorly differentiated phenotype and arises mostly in adolescents and young adults. Genetically, approximately 80% of the cases are characterized by a t(2, 13) or t(1, 13) chromosomal translocation, which generates the fusion oncoproteins PAX3-FOXO1 or PAX7-FOXO1 that work as mutant transcription factors (43, 44). ERMS is more common, usually affects children under the age of 10 years, and is for the most part associated with a favorable prognosis. Genomic landscape studies of RMS showed that ERMS has a higher mutation rate when compared to ARMS, as well as more frequent copy number variants and single nucleotide variants (45–47). Mutations identified include (among others) RAS isoforms, TP53, neurofibromin-1 (NF-1), PI3K catalytic subunit α (PIK3CA), β-catenin (CTNNB1), fibroblast growth factor receptor 4 (FGFR4), and F-box and WD repeat domain-containing 7 (FBXW7).

While the genomic homogeneity of ARMS would predict that its molecular features could be harnessed for therapeutic purposes, the PAX3-FOXO1 protein has remained therapeutically intractable (48). On the other hand, the genomic heterogeneity of ERMS highlights the challenge of finding a single target for therapeutic purposes. Using a variety of approaches, cell populations with CSC features have been reported for ERMS (49–52); the identification of ARMS CSCs has been more elusive and while a recent study showed that ARMS cells could form holoclones and spheres (53), no studies have reported *in vivo* functional assays for ARMS CSCs. Similar to what is observed in SS [below (54)], there is some thought that almost all PAX3-FOXO1^+^ ARMS tumor cells have stem cell characteristics–suggesting that ARMS is a stemness-disease, but this has yet to be demonstrated.

### Synovial sarcoma (SS)

SS is an aggressive neoplasm occurring in adolescents and young adults (aged 10 to 35 years), accounting for about 10% of all STSs (55). About 70% of cases develop metastases (56–58). SS is characterized by t(X;18)(p11;q11) (59), which generates an in-frame fusion of the synovial sarcoma translocation, chromosome 18 (*SS18*, also known as *SYT*) gene to the synovial sarcoma, X breakpoint (*SSX*) genes 1, 2 and, rarely, 4 (60–62). Whereas, SYT interacts with the SWI/SNF chromatin remodeling complex (63–65) to activate a host of transcriptional programs that control cell cycle, stem cell maintenance and differentiation signals (66–68), its fusion partner SSX co-localizes with the Polycomb repressor complex hampering its function (69). The resulting SS18-SSX fusion oncoprotein acts as an epigenetic modifier that is dependent upon cellular context (70), and drives SS pathogenesis (71–73). In line with fusion-driven sarcomas, SSs are genetically quiet, with no chromosomal abnormalities other than t(X;18) in the majority of cases. However, copy number gains are more common in adult patients and are typically associated with a poor outcome (74).

Skeletal muscle lineage precursors (but not differentiated myocytes) have been suggested as a cell of origin for SS, since conditional expression of the human fusion gene *SYT-SSX2* in Myf5-expressing murine myoblasts results in tumors with 100% penetrance (72). More recently, SYT-SSX2 forced expression in MSCs disrupted normal mesodermal differentiation, triggering a pro-neural gene signature via its recruitment to genes controlling neural lineage features (75). The authors also showed that SYT-SSX2 controlled the activation of key regulators of stem cell and lineage specification (75). Consistently, silencing of SYT–SSX induced terminal differentiation of SS cells into multiple mesenchymal lineages (osteogenic, chondrogenic and adipogenic types) (54). On the one hand, these data point to MSCs as a cell of origin of SS and suggest that deregulation of normal differentiation by SYT-SSX could constitute the basis for MSC transformation. On the other hand, they seem to also suggest that SS can develop in MSC precursors that are in a susceptible developmental stage. In the same work, Naka et al. showed that SS cell lines, similarly to SS clinical samples, contain a subpopulation of cells characterized by high levels of the pluripotency factors *SOX2, OCT4*, and *NANOG* and that exhibit *in vitro* self-renewal ability and *in vivo* tumorigenicity following xenografting (54).

### Fibrosarcoma (FS)

Adult type fibrosarcoma (FS) is a malignant tumor thought to arise from fibroblasts and is characterized histologically by undifferentiated spindle cells (76). Only a few studies point to the existence of CSCs within FSs. These studies identified a subpopulation of cells characterized by increased levels of *OCT3/4, NANOG, SOX2*, and *SOX10*, possessing stem-like characteristics such as self-renewal ability, proliferation and increased chemoresistance partly conferred by the overexpression of the multidrug resistance transporter MDR1 (77, 78). There are no published studies of CSCs in infantile FS, a pediatric malignancy associated with intermediate malignant rarely metastasizing tumor characterized by the *NTRK3–ETV6* translocation resulting from t(12, 15) (79).

### Malignant peripheral nerve sheet tumor (MPNST)

MPNSTs account for about 5-8% of all STSs and can occur sporadically after radiotherapy or can arise in the neurofibromatosis type 1 (NF1) syndrome (80). The *NF1* gene, located on the long arm of chromosome 17 (17q11.2), encodes for the 220kDa protein neurofibromin. NF1 syndrome is characterized by mutation-induced inactivation or more rarely complete germline loss of one *NF1* allele that often leads to either dermal or plexiform benign neurofibromas. The latter neurofibroma subtype, arising in nerve plexuses or deep large nerves, occurs following *de novo* somatic mutations or inactivation of the other NF1 allele specifically in the Schwann cell lineage and can undergo malignant transformation in MPNSTs (81). Patients with NF1 can develop other types of pediatric tumors such as pheochromocytomas, RMS, LMS, and juvenile myelomonocytic leukemia (82). In addition, inactivating mutations of *NF1* have been reported in adult tumors including brain, lung and ovarian cancers and in melanomas (83). Neurofibromin inhibits RAS signaling through its RAS GTPase-activating protein (GAP) domain, thus working as a tumor suppressor (84). In agreement, the RAS pathway is constitutively over-activated in MPSNTs (85). Although neurofibromin is a member of the large RAS-GAP family proteins, it is the only one linked to a tumor predisposition syndrome when mutated. However, accumulating genomic abnormalities in tumor suppressors or oncogenes have been suggested to be responsible for the progression from benign plexiform neurofibromas to MPNSTs. Loss of *TP53* and *CDKN2A* are common in MPNSTs (86, 87). *CDKN2A* encodes for both p19^ARF^ and p16^INK4A^ and thus its inactivation can affect both p19^ARF^-MDM2-p53 and p16^INK4A^-Cyclin D-RB pathways leading to uncontrolled proliferation. Even *RB1* loss can be seen in about 25% of MPNSTs (88).

Either precursors of or postnatal Schwann-derived cells, the source of myelinating glial cells of the peripheral nervous system, seem to be the cell of origin of MPNSTs (82, 89, 90). Gene expression studies showed that MPNSTs exhibit deregulation of tumor-specific gene clusters belonging to Schwann cell development regulators, including downregulation of *SOX10*, which promotes the specification of Schwann progenitors and their maturation and myelin production (91) and upregulation of *SOX9*, which is involved in neural crest stem cell survival (92, 93). Putative CSCs of MPNSTs expressing stemness genes have been recently established under specific conditions from human cell lines and primary tumors (94). These cells were characterized by high levels of the neural lineage genes *NESTIN* and *NGFR*, and the stemness markers *OCT4, NOTCH4, SOX2*, and *SOX9* as assessed by qPCR, but also by the expression of stem surface markers by flow cytometry, and were shown to give rise to tumors resembling human MPNSTs when they were injected subcutaneously in immunodeficient mice.

### Leiomyosarcoma (LMS)

LMS accounts for about one quarter of all soft tissue tumors. It is extremely rare during infancy and childhood, occurring most commonly in middle-aged individuals. LMS has a complicated histopathological classification and a different clinical behavior depending on the location within the body (95). LMS is characterized by a high degree of genomic instability, with non-recurrent aberrations at the chromosomal level. The most common regions of chromosomal loss have been identified in 10q and 13q, where the phosphatase and tensin homolog (PTEN) and the retinoblastoma 1 (RB1) tumor suppressor genes, respectively, reside (96–98). In addition, PTEN point mutations (99, 100) as well as constitutive hyperactivation of PI3K/AKT pathway (101), have been detected in LMS, suggesting that loss of PTEN might contribute to the initiation or progression of LMS. Epigenetic changes could also contribute to LMS. For instance, Roncati et al. showed that methylation-dependent silencing of *CDKN2A*, which is associated with decreased expression of both p16^INK4A^ and p14^ARF^ and with higher activity of MDM2 and CDK4/CDK6, results in LMS progression (102). Additionally, treatment with the HDAC inhibitor vorinostat in combination with a DNA demethylating agent such as decitabine allowed overcoming the resistance to cell death induction due to promoter methylation of apoptotic genes in uterine LMS (103).

Recent findings suggest a mesenchymal stem cell origin for LMS. By using Cre-Lox technology to generate murine MSC cultures knock-out for Trp53 and Rb1 alone or in combination, Rubio et al. showed that *Tp53*^−/−^ mouse adult MSCs underwent *in vitro* transformation and developed LMS-like tumors *in vivo* when injected as xenografts in immunodeficient mice (104). A microRNA (miRNA) signature distinctive of MSCs includes several components of the miR-17-92 cluster, and appeared downregulated during MSC differentiation into SMCs while up-regulated in uterine LMS, supporting the hypothesis that LMS is a mesenchymal stem cell-related malignancy (32). In testicular LMS, a subpopulation of cells with stem-like characteristics was described (105). These cells showed high tumorigenic potential and the capacity to re-derive the original parental tumor in immunodeficient mice.

### Liposarcoma (LPS)

LPS is also one of the most common STS, accounting for at least 20% of all adult sarcomas. The World Health Organization classifies LPS into four main subtypes: 40–45% well differentiated (WD-LPS), 15–25% dedifferentiated (DD-LPS), 30–35% myxoid/round-cell (MR-LPS) and about 5% undifferentiated high-grade pleomorphic liposarcomas (P-LPS) (106). The histological subtypes reflect both clinical behavior and prognosis (107). WD-LPS occurs most frequently in the retroperitoneum and limbs, rarely metastasizes and shows low recurrence rates. By contrast, DD-LPS is more aggressive, with a metastatic rate of 15–20% and a worse prognosis. Both WD-LPS and DD-LPS can be distinguished from other adipocytic neoplasms based on the amplification of the chromosome region 12q13-15, in which *MDM2, CDK4* and *SAS* genes reside (108). MR-LPS is characterized by the appearance of spindle to oval-shaped cells in a myxoid stroma (109) and has a predilection for the limbs, with abdomen and bones as typical metastatic sites (110, 111). MR-LPS harbors the chromosomal translocation t(12, 16)(q13;p11) that results in a fusion gene arrangement between *FUS* and the *C/EBP* homologous protein (CHOP, also known as DDIT3 or GADD153) and appears to constitute the primary oncogenic event in MR-LPS (112). P-LPS is a rare tumor of adulthood and can be distinguished from the other subtypes by the presence of pleomorphic lipoblasts. It occurs most commonly in soft tissues of the extremities and is associated with pulmonary metastases (113).

Previously, adipose-derived stem cells (ASC) were proposed as a cell of origin of LPS (114). In support of this, Rodriguez and colleagues showed that the expression of a *FUS-CHOP* transgene in Trp53-deficient mouse ASCs was able to shift the tumor phenotype toward LPS-like tumors (115). Consistently, the expression of FUS-CHOP in murine MSCs resulted in the development of tumors resembling LPS with features such as intracellular lipid accumulation, presence of lipoblasts with round nuclei, and an adipocyte differentiation block (116). In the human setting, *FUS-CHOP* has been reported to cooperate with other oncogenic hits to block the differentiation potential of bone marrow-derived MSCs toward adipocytes, and to transform them into LPS cells resembling the myxoid subtype (117). Matushansky and colleagues linked adipocyte differentiation from human MSC to all LPS subtypes, in dependence of their maturation status (33). They propose that additional secondary mutations could lead to morphologically diverse tumors arising from the same stage of transformation (33).

Using a LPS xenograft model, Stratford et al. identified a small (0.1–1.7%) fraction of cells characterized by a stem-like phenotype (CD133^+^/ALDH^+^). This putative CSC population was able to self-renew *in vitro*, differentiate into mature adipocytes and be highly tumorigenic in nude mice (118). Recently, LPS-like tumor xenografts were generated in immunocompromised mice from subcutaneous injection of murine induced pluripotent stem cells (miPSCs) that were cultured in conditioned media from the Lewis lung carcinoma (LLC) cell line, which secretes tumor-derived extracellular vesicles [the resulting cells were termed miPSC-LLCev] (119). Cells derived from the LPS-like xenografts were characterized by CSC features including self-renewal ability, increased expression of Sox2, Nanog and Klf4, the capacity to generate secondary tumors resembling the original histotype of the primary ones, and to disseminate into the mesentery of abdominal cavity. When cultured as spheres, the miPSC-LLev cells were also able to differentiate into adipocytes under appropriate conditions, suggesting phenotypic heterogeneity (119).

### Undifferentiated pleomorphic sarcoma (UPS)

UPS is the most common STS in the elderly. In the 1960s this malignancy was thought to arise from histiocytes and thus was named “malignant fibrous histiocytoma.” It was later proven that this tumor was not a true histiocytic malignancy and it was renamed pleomorphic fibrosarcoma, or undifferentiated high-grade pleomorphic sarcoma (UPS) (120). UPS cells have variable morphology without a hint of differentiation (121). Recently, rare gene fusions involving *PRDM10* were identified in UPS tumors (122). Li et al formed pleomorphic sarcomas in immunodeficient mice out of a transformed culture of bone marrow stromal cells (30). Martinez et al were also able to engineer a model of UPS out of a mutated human bone-marrow derived MSCs, proving that MSCs are most likely the origin this sarcoma (123, 124). Rubin et al. were able to generate UPS through the loss of Tp53 in combination with Ptch1 in Myf6-expressing muscle cells. Interestingly, however, when these cells were depleted only of Trp53, they formed ERMS, suggesting a common progenitor (125). Wang et al. isolated a subpopulation of cells in UPS that exhibits stem-like properties identified as a “side population” by flow cytometry (126).

## Isolation and characterization of CSCs in STS

**Figure 1:**
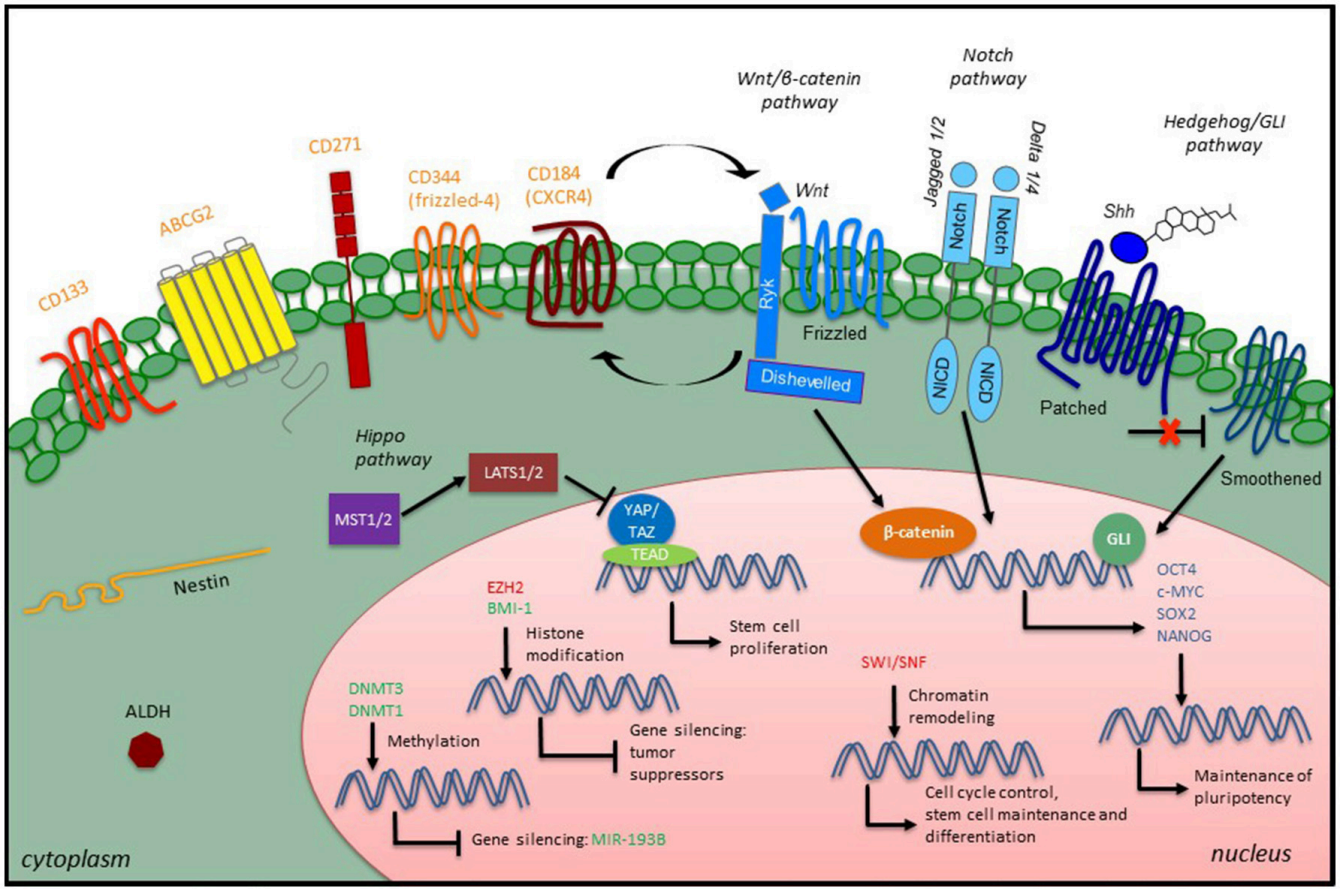
Overview of CSC characteristics and signaling in STS. STS CSCs express specific stem cell surface markers (orange), which have been used as CSC identifiers, along with some intracellular markers such as the intermediate filament Nestin or the enzyme ALDH. Developmental signaling pathways play a role in the CSC phenotype by promoting the expression of embryonic transcription factors (blue). Epigenetic modulators (green, confirmed modulators; red, putative modulators) also participate in the CSC phenotype through different mechanisms: maintenance of existing methylation patterns or *de novo* methylations at CpG islands, histone modification and chromatin remodeling.

Successful and specific investigation of CSCs is a prerequisite for better understanding of the molecular mechanisms underlying STS initiation, progression, relapse, metastasis and resistance to therapies. Currently, evaluation of CSCs is achieved through both *in vitro* and *in vivo* approaches, including (i) generation of non-adherent “sarcosphere” cultures under serum-free conditions; (ii) flow cytometry sorting based on expression of CSC-specific surface markers; (iii) assessment of aldehyde dehydrogenase (ALDH) activity through the ALDEFLUOR (Stemcell Technologies) assay; (iv) detection of a side population identified by exclusion of Hoechst 33342 dye; (v) xenograft tumor initiation of the ability of a low number of cells in immunodeficient mice, and (vi) reprogramming of cancer cells back to pluripotency (127–129). Remarkably, since none of these methods alone is enough to identify CSCs unequivocally within a tissue, the use of several markers and properties in combination could be helpful to better define the CSC phenotype in these tumors. The stem cell markers and assays that have been used to identify, isolate and characterize potential STS CSCs are summarized in Table 1. An overview of STS CSCs including their characteristics and signaling is provided in Figure 1.

**Table 1:** List of soft tissue sarcoma (STS) subtypes and the stem cell markers and assays that have been used to investigate their cancer cell stemness.

**Subtype**	**Stem cell markers and assays**
Rhabdomyosarcoma (RMS)	CD133 CD184 (CXCR4) ABCG2 Nestin ALDH Sphere formation
Synovial sarcoma (SS)	CD133 CD271 CD344 ABCG2 ALDH Sphere formation Side population/dye efflux
Fibrosarcoma (FS)	CD133 CD271 ABCG2 ALDH Sphere formation
Malignant peripheral nerve sheet tumor (MPSNT)	CD184 (CXCR4) Nestin Sphere formation
Leiomyosarcoma (LMS)	CD184 (CXCR4) CD271 CD344 Sphere formation Side population/dye efflux
Liposarcoma (LPS)	CD184 (CXCR4) CD271 ALDH Sphere formation
Undifferentiated pleomorphic sarcoma (UPS)	Side population

### Three-dimensional cell cultures

First used by Reynolds and Weiss to isolate stem cells of neural origin (130), the 3D model enables cells to grow in all dimensions, thus mimicking the interactions between cells of interest and the microenvironment in a given tissue (131). A single-cell suspension is grown in low-density conditions to avoid cell aggregation, and in defined serum-free media supplemented with specific growth factors (epidermal growth factor, and basic fibroblast growth factor), in ultra-low attachment plates (132). In these conditions, cells can proliferate to form non-adherent, floating spheres, which in turn can be dissociated to allow secondary and tertiary sphere formation. Each sphere consists of a small percentage of self-renewing cells and a large percentage of progenitor cells at various stages of differentiation (133). In the last few years, sphere culture techniques have been employed to allow CSC enrichment in STSs, including both ERMS and ARMS (50, 53), FS (77), SS (54, 77), MPNSTs (94), LMS (105), and LPS (118, 119).

### Stem surface markers

The first idea for CSCs isolation based on the expression of certain surface markers came with the identification of a small subset of leukemic stem cells with a CD34^+^CD38^−^ phenotype. When these cells were transplanted into non-obese diabetic/severe combined immunodeficient (NOD/SCID) mice, they were able to initiate acute myeloid leukemia with the same heterogeneity of the primary cancer, whereas the most abundant subset of CD34^+^CD38^+^ was ineffective (11). These results stimulated many researchers to isolate CSCs from heterogeneous cell populations of STS through fluorescent-activated cell sorting (FACS) of cells expressing specific stem surface markers, alone or in combination. Following is a list of cell surface markers thought to have roles in stemness.

**CD133** (Prominin-1) is a glycosylated protein involved in topological organization of the cell membrane (134). CD133 has been shown to mark a subpopulation of tumor cells characterized by high levels of stemness genes, higher clonogenicity and *in vitro* self-renewal, and increased *in vivo* tumorigenicity than the CD133- subpopulation in several STS, including SS (135–137), RMS (49, 50, 52, 138) and FS (77, 78, 136). Similarly, LPS putative CSCs, prospectively isolated by FACS of the stem surface marker CD133 and of ALDH activity, were shown to produce tumors at limiting cell dilution more efficiently compared to the other sorted subpopulations (118). In MPNST, CD133^+^ cells were able to self-renew, yielding more spheres and proliferating faster compared to the CD133^−^ cells, and to give rise to tumors resembling the original heterogeneous tumor when injected at low number (94). In LMS, She et al. found that CD133 expression positively correlates with tumor size, mitotic counts and histological grade in primary retroperitoneal LMS, proposing it as prognostic marker (139).

**CD184** (chemokine receptor type-4, CXCR4) is a seven transmembrane chemokine receptor normally expressed on immune cells, but also on embryonic stem cells (ESCs) (140) and MSCs (141). Recently, CD184 has been identified as a SS-initiating surface marker. By using sphere formation assays, the authors enriched for a CSC subpopulation that was characterized by high levels of CXCR4. These cells exhibited higher tumor initiation potential after serially transplantations into NOD/SCID mice compared to their CXCR4^−^ counterpart and were able to recapitulate the phenotype observed in the original tumor (142). Interestingly, CXCR4 was found highly expressed on the surface of ARMS cells, where it correlates with unfavorable primary sites, advanced stage, decreased overall survival and bone marrow involvement (143, 144), and was also used as a prognostic marker for MPNSTs, LMS, LPS and FS (145). However, the above-mentioned studies did not determine whether CD184 is associated with a CSC phenotype in these STS.

**CD271** (low-affinity nerve growth factor receptor), is expressed in neural crest tissue and suggested to be a CSC surface marker in SS, FS, LMS and LPS (146).

**CD344** (frizzled-4), a neuronal stem cell marker that plays important roles in vascular development of the retina and inner ear, has been shown to identify a tumor cell subpopulation with increased capacity for proliferation and sarcosphere formation and resistance to doxorubicin in LMS and SS cells (146).

**ABCG2** (ATP binding cassette G2) has been used to isolate a subpopulation of CSCs with increased drug resistance in SS and FS (77, 78). ABCG2 expression has been associated with shortened survival in RMS patients (147).

**Nestin** is an intermediate filament protein first identified in stem cells of neuroepithelial origin. It is expressed in several cell types during development, including neural crest cells and myocytes. It was found upregulated in tumor cell spheres derived from MPNSTs compared to their corresponding adherent cells (94) and overexpressed in RMS (148).

### ALDH activity

In addition to the cell surface markers described above, a few specific intracellular enzymes and their activity can be utilized to identify CSCs. Among them, the detoxifying enzyme aldehyde dehydrogenase (ALDH), which is highly expressed in normal stem cells (149, 150), has been recently implicated in therapy resistance and tumor recurrence (151–154). Detection of ALDH activity is captured through the ALDEFLUOR assay, an enzyme-based assay thought to specifically detect the ALDH isoform ALDH1A1 (155). ALDH proteins have been used as markers of CSC identification in many STSs including ERMS (51), LPS (118), FS and SS, in which their expression correlates with higher proliferation and clonogenicity, and is associated with increased drug resistance (156).

### Side population

The side population phenotype (SP) was first defined in hematopoietic cells (157, 158). SP cells show limited intake or active extrusion of the fluorescent dye Hoechst 33342 as a consequence of increased expression of the ABC transporter ABCG2 (159–161). SP cells can be isolated by flow cytometry based on the absence of accumulation of Hoechst dye, and have been used to enrich for CSCs in various cancers, including sarcomas (162, 163). Indeed, the SP phenotype has been shown to correlate with high tumorigenicity in immunocompromised mice, the ability to repopulate both the SP and the non-SP fractions (164, 165) and resistance to therapy (166–168)-all established criteria for CSCs. In the context of STSs, Alman's group was the first to identify a SP fraction within human LMS and SS through Hoechst dye staining. The size of this SP appeared to positively correlate with the tumor grade, although it is unclear whether the SP fraction isolated in this study reflected a population of cells enriched in CSC features such as self-renewal ability and higher *in vivo* tumorigenicity compared to non-SP cells (169). By contrast, Sette et al. demonstrated that the subpopulation of testicular LMS stem-like cells characterized by a SP phenotype showed enhanced ability to extrude doxorubicin and high clonogenicity in limiting dilution assays (105).

### Limiting dilution *in vivo*

The most stringent method to define the frequency of CSCs *in vivo* is the limiting dilution cell transplantation assay (LDA). In this assay, tumor cells are transplanted at defined, decreasing doses into animals and tumors allowed to develop over time. At analysis, the percentage of animals that develop (or do not develop) tumors is used to determine the number of tumor cells with self-renewal capacity (170, 171). These “LDA frequencies” are calculated using a web-based tool called ELDA (extreme limiting dilution analysis), which is the first software for limiting dilution analysis that delivered useful confidence intervals for all LDA cell subpopulations, even those with no (or complete) response (172). *In vivo* LDA must be performed to confirm that a defined marker enriches for CSC activity, and must be done with both the positive and negative fractions. It is important to highlight that the *in vitro* sphere-forming assay does not constitute a surrogate for the *in vivo* LDA, and can only complement, rather than replace, it. By using a Trp53-null mouse model of breast cancer, Zhang and colleagues identified a cell subpopulation characterized by high levels of CD24 and CD29 using *in vitro* LDA and subsequent transplantation *in vivo* (173). However, to date LDA has been performed only for few STS tumors *in vivo*, and further studies are required to confirm the true nature of marker-sorted CSCs in STSs.

### Reprogramming mechanisms potentially involved in CSCs in STS

Similar to normal cells, in which reprogramming toward a pluripotent state can be achieved by nuclear transfer, blastocyst injection, or by applying induced pluripotent stem cell (iPSC) technology [forced expression of a specific panel of TFs such as Oct4, Sox2, Klf4, and Myc (174, 175)], human cancer cells can also be reprogrammed. Hochedlinger and colleagues showed that introduction of nuclei derived from mouse melanoma cells into enucleated oocytes induced the establishment of an ESC line from blastocysts with the potential to generate teratomas (176). Alternative methods to reprogram cancer cells include: the transfection of cancer cells with the family of micro-RNA miR-302 that is highly expressed in ES cells, thus generating pluripotent stem-like cells with both self-renewal and multipotency properties (177, 178); the use of small chemical molecules to enrich for cancer stem-like cells endowed with increased *in vitro* tumor-sphere formation and *in vivo* tumorigenic abilities (179); and the application of the iPSC technology to primary tumor cells to successfully convert several cancer types into induced-pluripotent cancer stem cells (iPCSCs) (176, 180–182). These iPCSCs resemble the ES cell state at both epigenetic and transcriptional levels and repress the reprogrammed cancer genome in the pluripotent state, constituting a live cell model for studying cancer progression (183).

#### Stemness signals

It is known that adult SCs and CSCs both express key evolutionarily conserved developmental pathways such as Wnt/β-catenin, Notch, Hedgehog and Hippo, which play pivotal roles in regulating stemness and differentiation (184–189). In fact, aberrant stemness signaling has been related to tumorigenesis, as deregulation of these pathways in adult SCs can lead to unchecked cell proliferation and aberrant differentiation in a tissue-specific manner. Besides, their reactivation in bulk tumor cells plays critical roles in cancer plasticity, by inducing a CSC phenotype, to promote EMT and to enhance drug resistance (190–192). However, the final goal is the alteration of gene expression patterns through the induction of embryonic TFs such as OCT4, NANOG, SOX2, KLF4, and MYC, which are part of a transcriptional network in which they regulate each other and affect chromatin remodeling (193–195). The existence of a link between reprogramming mechanisms and the stem cell TF network is supported by the revolutionary study of Yamanaka and colleagues, showing that lineage committed cells can be reprogrammed to an induced pluripotent state after the introduction of Sox2, Oct4, Klf4, and Myc (175). As such, a stemness signature is seen more often in less differentiated cancers with worse clinical outcomes (196, 197).

##### Hedgehog pathway

The Hedgehog (HH) pathway is a signaling network that plays a crucial role during organogenesis in the developing embryo, mainly by modulating genes involved in stem cell fate determination (198). Similarly, it is important for the existence of CSCs, as it is believed to support the CSC phenotype by driving the expression of stemness-related genes, including Oct4, Sox2, Bmi1, and Nanog (199). However, few studies are currently available about its role in CSCs of STS. In 1996, Hahn et al described a critical role for HH pathway in ERMS (200). Later studies confirmed then its activation in ERMS, its role in promoting self-renewal of ERMS CSCs (201, 202), and its association with a poor prognosis (203). The HH pathway has been proven to be hyperactivated also in ARMS, although it appears mainly activated in translocation-negative ARMS compared to translocation-positive ones (202), and its upregulation has been associated with a poor prognosis (204). A recent study showed HH pathway activation in cells contained in CSC-enriched structures including holoclones and spheres (53). In UPS, the HH pathway has been linked to the maintenance of self-renewal and proliferation of a subpopulation of putative CSCs characterized by a SP phenotype (126).

##### Hippo pathway

The Hippo tumor suppressor pathway plays key roles in tissue homeostasis and repair by regulating stem cell proliferation and expansion (203). It consists of cytoplasmic kinases (MST1/2 and LATS1/2) that act as tumor suppressors by restraining the activity of the transcriptional co-activators yes-associated protein (YAP1)/transcriptional co-activator with PDZ-binding motif (TAZ, also known as WWTR1) via phosphorylation, hence preventing their nuclear localization and activation of TEA domain (TEAD) family members transcription factors. Dysregulation of the Hippo pathway has been linked to cancer development, including STSs (205–208). Besides, increased expression of YAP/TAZ has been correlated with the acquisition of cancer stem cell traits that lead to EMT, drug resistance and metastasis (209). Recent evidence points to the role of the Hippo pathway in maintaining CSCs in STS. For instance, Linardic and co-workers identified a novel NOTCH-YAP1-SOX2 circuit critical for maintaining stem cell plasticity in ERMS (210). The same group found expression of TAZ in ARMS, in which it supports stemness and promotes drug resistance (211).

##### Notch pathway

The Notch pathway regulates cell fate determination mainly of stem and/or progenitor cells during embryonic development of organs such as pancreas, bones, muscles, heart and the nervous system (186, 212, 213), and its role in CSC establishment and maintenance has been reported in a wide range of human cancers (214, 215). The Notch pathway was found deregulated in some STS. For instance, both ERMS and ARMS show significant upregulation of the pathway, which has been shown to affect motility and invasiveness of both RMS subtypes (216). However, its importance in RMS CSC self-renewal and differentiation has been reported only for the ERMS subtype (217). The Notch pathway was found hyperactivated also in SP cells of UPS compared to non-SP cells, pointing toward a critical role in in maintaining CSC self-renewal and proliferation (126). In SS, Notch pathway components NOTCH1, JAG1 and the transducin-like enhancer of split (TLE)-1 were found overexpressed, although any association with the CSC phenotype of SS has not been yet reported (218).

##### Wnt/β-catenin pathway

The Wnt/β-catenin pathway plays roles in cell fate determination, cell proliferation and migration during embryogenesis. After development, it participates in preserving homeostasis in different organs, mainly those who rely on the function of stem cells (184, 185). Wnt/β-catenin signaling is involved in MSC self-renewal and differentiation (219) and it appears aberrantly expressed in a variety of CSC settings (220), although its role as oncogenic influence is still debated since its recent association with a tumor suppressive effect (221, 222). As an example, in ERMS some researchers observed inhibition of the Wnt/β-catenin pathway (223), whereas others identified opposing roles for the canonical and non-canonical pathway in regulating ERMS self-renewal and differentiation, as the canonical pathway plays a tumor-suppressor role whereas the non-canonical pathway an oncogenic one (224). By contrast, Wnt/β-catenin pathway has not been extensively studied in ARMS, although Kephart et al. found that an inhibitor of the Wnt/β-catenin pathway secreted frizzled related protein 3 is upregulated in PAX3-FOXO1+ human ARMS cells, suggesting a tumor-suppressive role for Wnt/β-catenin signaling (225).

In SS, Barham et al. provided evidence that the fusion protein SYT-SSX2 activates Wnt/β-catenin signaling through its nuclear reprogramming function, using a combination of SS cell cultures, xenografts and a *SYT-SSX2* transgenic mouse model. In this study, inhibition of the Wnt pathway with small molecule CK1α activators induced SS growth arrest and, importantly, reversed the myogenesis block induced by the fusion oncoprotein by downregulating many of the Wnt/β-catenin targets involved in the embryonic program, including pluripotency factors, differentiation blockades, embryonic lineage determinants, homeobox, and forkhead box factors (226), suggesting that targeting this pathway may a rational approach in patients with SS (227, 228).

In MPNSTs, a forward genetic screen using the Sleeping Beauty mutagen transposon approach *in vivo* revealed the over-activation of Wnt signaling, exemplified by overexpression and enhanced activity of β-catenin due to both decreased levels of the destruction complex and up-regulation of R-spondin2, a secreted ligand that can stimulate Wnt signaling. Data were confirmed in murine models of MPNST and in primary samples from patients, and the translational relevance was verified from the evidence that the blockade of this pathway affected the tumorigenic properties of tumor cell lines *in vitro* and *in vivo*. Notably, simultaneous inhibition of Wnt signaling and mTOR pathway, the latter over-activated in this tumor, showed synergistic effects suggesting a path to intervention (229). It has been proposed that Wnt/β-catenin signaling activation could be also triggered by an autocrine loop via the activation of the CXCR4 receptor, which is overexpressed on the surface of MPNST cells, resulting in the inactivation of GSK3β that mediates β-catenin destabilization (230).

#### Epigenetic alterations

Alterations of the epigenetic machinery are considered critical for CSC formation and persistence. In both embryonic and adult SCs, epigenetic processes modulate the transcriptional programs to regulate the balance of self-renewal vs. differentiation. Thus, undifferentiated SCs express high levels of TFs OCT4, SOX2, and NANOG, which work to ensure the maintenance of self-renewal and pluripotency through their co-localization on specific regulatory regions (231, 232). During the differentiation program, genes that are associated with pluripotency and self-renewal become silenced, whereas tissue-specific genes are turned on (233). Interestingly, developmental and lineage-committed genes are often present within “bivalent” chromatin domains (promoters and enhancers) containing both repressive (H3K27me3 or H3K9me3) and permissive (H3K4me3 or H3K4me1) histone marks (234–237). These genes are repressed even if polymerase II is present on bivalent promoters/enhancers to allow rapid activation (236, 238, 239) by safeguarding differentiation. These bivalent regions appear to be enriched for binding sites for at least one of the pluripotency TFs mentioned above and can be poised for transcriptional activation or repression during both developmental and differentiation processes to respond quickly to developmental cues (238, 240–242).

Some cancer cells have bivalently marked genes that correspond to those in embryonic SCs, but a remarkable number of regions that are bivalently marked in SCs are frequently hypermethylated and thus completely silenced in cancer cells (243, 244). Local DNA hypermethylation at tumor suppressor genes or genes associated with differentiation has been shown to predispose precancerous cells to oncogenic transformation and CSC establishment (243, 244). Mechanistically, many tumor cells show aberrant activation of the DNA methyltransferases DNMT1 and DNMT3, which are involved in the maintenance of existing methylation patterns or in the *de novo* methylation at CpG islands, respectively. DNMT hyperactivation is also required for the maintenance of the CSC subpopulations (245).

In the context of STSs, increased expression of DNMT1 in LPS (compared to fat) results in miR-193b downregulation by promoter methylation (246). Expression of miR-193b has been linked with adipogenesis in adipose-derived SCs and with increased apoptosis in LPS cells, as miR-193b mimetics were able to inhibit LPS xenograft growth *in vivo* (246). However, further studies are required to clarify whether DNMT1 might play a role in the establishment of CSCs in LPS. Similarly, in ERMS DNMT3B appears to be important for the maintenance of a less differentiated phenotype, since its depletion reverses cell cancer phenotype by rescuing the myogenic program (247). In uterine LMS, treatment with the HDAC inhibitor vorinostat in combination with a DNA demethylating agent such as decitabine allowed overcoming the resistance to cell death induction due to promoter methylation of apoptotic genes (103). Given that resistance to chemotherapeutics represents an essential characteristic of CSCs, it is possible that DNA methylation might favor a CSC phenotype.

The process of CSC reprogramming has been also correlated to histone modifications. Thus, it is not surprising that epigenetic modifiers constitute the most altered genes in both solid cancers and hematological malignancies (248). For example, enhancer of zeste homolog 2 (EZH2), the catalytic subunit of the polycomb repressive complex PRC2, has been found overexpressed in several tumors, in which it contributes to H3K27me3-mediated silencing of tumor suppressor genes, besides promoting a self-renewal transcriptional program that allows CSC expansion (249–253). Similarly, upregulation of a key subunit of the PRC1 complex, BMI1, has been shown to favor the reprogramming toward a CSC phenotype through the repression of tumor suppressor pathways in tumor-initiating cells (254, 255).

Except for BMI1, which has been reported to be highly expressed in fractions of CD133^+^ SS cells, characterized by increased clonogenicity, self-renewal, and *in vivo* tumor formation (137), no association between histone modifications and CSC phenotype has been demonstrated to date in STS. However, studies showed altered expression of the epigenetic modifier EZH2 in ERMS and SS, which has been related to the survival of cancer cells and to the maintenance of a less differentiated and more aggressive phenotype, suggesting pharmacological inhibition of EZH2 as adjuvant differentiation therapy (256–258). Also, a direct involvement of PRC2 components in the progression from neurofibroma to MPNST has been demonstrated showing that, surprisingly, EZH2 works as a tumor suppressor, and the detection of the loss of H3K27 trimethylation has entered the clinical practice to help in the diagnosis of MPNST (83).

Three well characterized ATP-dependent chromatin remodelers (SWI/SNF, ISWI, CHD) have also been also implicated in tumor initiation. Loss of the SWI/SNF complex, for instance, plays an important role in sarcomagenesis (259–261). Together, these data indicate that alterations in chromatin status may represent a key step for CSC formation and maintenance, by inducing the activation of several stemness signals in differentiated cancer cells. In SS, the SS18-SSX fusion protein has been shown to compete with SS18, a normal subunit of the SWI/SNF complex, for the assembly into the complex, thus reverting the H3K27me3-mediated repression at the *Sox2* locus (262). In RMS, several lines of evidence suggested that alteration of SWI/SNF components might help to maintain tumor cells in a less-differentiated state. Indeed, the activation of the ATPase subunit of the SWI/SNF chromatin remodeling complex BRG1, for instance, provided an open chromatin conformation for the induction of myogenin in ERMS cell lines (263). Likewise, silencing of the SWI/SNF complex subunit BAF53a, which has been found hyper-expressed in primary RMS tumors compared to normal muscle, increases the expression of myogenic markers contributing to the differentiation program (264). Interestingly, CHD4 was identified as an important epigenetic co-regulator of PAX3-FOXO1 activity in ARMS. Together, both of these proteins bind to the regulatory regions of PAX3-FOXO1 target genes (265). All together, these data emphasize the necessity to address the requirement of these epigenetic modifiers in the maintenance of a stem-like phenotype in STSs.

## Challenges and future directions

The role of CSCs in tumorigenesis and the ability to therapeutically target their vulnerabilities will continue to be important for all cancer types. However, since STSs are less common than—for example—cancers of the breast, colon, lung, prostate, and melanoma, and exhibit enormous cellular and molecular heterogeneity, information regarding stemness in STS and how to apply it therapeutically will lag. On the other hand, because of the unique characteristics of STSs, there may be unanticipated opportunities for investigation. For example, since STSs are all soft tissue cancers of mesenchymal origin, can we identify conserved CSC signatures spanning STSs that can be exploited for therapy? Since many STSs have unique signature translocations that drive their tumorigenesis, can we compare and contrast the impact of the encoded oncogenic fusion proteins on CSC stemness and identify commonalities to target? It is likely that there is not a “one-size-fits-all” CSC for STSs, and that translocation-positive STSs with their more homogeneous genomes may need to be studied separately from translocation—negative STSs in order to understand how and when the stochastic and hierarchical models of CSCs apply.

There remain many gaps in the STS CSC field. Some of these gaps are technical—for example, can we standardize our definition of CSC, and consistently apply the most stringent criteria for “stemness,” which is the ability for a small number of tumor cells to give rise to an STS, rather than rely on descriptions of stemness TF or cell surface marker expression? On the other hand, some of these gaps are conceptual. Because of the sheer number of STSs subtypes and the intrinsic complexity of a heterogeneous tumor, it has not been possible to undertake a comprehensive and systematic investigation of other forces that impact cancer cell stemness in STSs, such as the microenvironment, the CSC niche, the role of immunoediting, mechanical cellular forces, and so forth (26, 266). Bridging these knowledge gaps will take time and coordinated effort between fields including but not limited to cancer biology, bioinformatics, mathematics, bioengineering, immunology, and evolutionary biology.

Regarding future directions, two fields in particular are rapidly changing and having an immediate impact on STS biology and therapy: epigenetics and immunotherapy. Knowledge of epigenetic circuitry in both SC and CSC is increasing, and many of the involved proteins have druggable moieties (267). Can these moieties be evaluated in STS basic and preclinical studies? Once we identify these moieties, can we then understand patterns of treatment resistance, whereby one CSC epigenetic circuit might compensate for another? Knowledge and application of immunotherapy has also revolutionized the treatment of cancer at the CSC level, including via monoclonal antibodies, checkpoint modulators, and CAR-T approaches (268). For example, targeting CSC markers is currently being attempted via CAR-T cells against CD133 (clinicaltrials.gov NCT02541370, NCT03423992, NCT03473457). Caution must be maintained, however, since in some carcinoma xenograft studies even CD133^−^ cells can initiate tumors (269). Nevertheless, with transdisciplinary approaches, we should have confidence that knowledge of STS cancer stem cell biology will also progress and lead to improve patient outcomes.

## Author contributions

CL, RR, SP, and KG: Conceived review; KG and SP: Wrote the paper; CL and RR: Edited the paper.

### Conflict of interest statement

The authors declare that the research was conducted in the absence of any commercial or financial relationships that could be construed as a potential conflict of interest.
